# The microbiome and gut–lung axis in nontuberculous mycobacterial pulmonary disease

**DOI:** 10.1371/journal.ppat.1013603

**Published:** 2025-10-17

**Authors:** Ria N. Thompson, Antje Blumenthal, Mark Morrison, Rachel M. Thomson

**Affiliations:** 1 Greenslopes Clinical Unit, Faculty of Health, Medicine & Behavioural Science, The University of Queensland, Brisbane, Australia; 2 Frazer Institute, The University of Queensland, Translational Research Institute, Brisbane, Australia; 3 Department of Gastroenterology and Hepatology, Princess Alexandra Hospital, Brisbane, Australia; 4 Gallipoli Medical Research, Greenslopes Private Hospital, Brisbane, Australia; Carnegie Mellon University, UNITED STATES OF AMERICA

## Abstract

**Nontuberculous mycobacterial pulmonary disease (NTM-PD)** is increasingly recognised as a significant global health concern. It is characterised by a highly heterogenous clinical course and remains poorly understood, from host susceptibility to disease pathophysiology, and is notoriously difficult to treat. Recent advances highlight the **microbiome** as a critical modulator of host physiology, with site-specific ‘microbiota’ influencing the delicate balance between health, infection and disease. While microbial populations vary across discrete anatomical sites, there is a growing recognition that they are interconnected. For example, gut microbes can influence immune cell functions in the lung via the **gut–lung axis (GLA).** Drawing parallels with other related chronic respiratory diseases, it is hypothesised that microbiota–host-interactions shape susceptibility and manifestation of NTM-PD. This review synthesises current knowledge of some key host susceptibility factors in NTM-PD, and their potential interactions with host microbiota. With only recently emerging studies, we explore the potential role of the GLA in NTM-PD, given its promising links to microbial communities and immunological and metabolic pathways. We assess the limited, but growing body of research on the lung microbiota in NTM-PD and evaluate the small number of studies on faecal microbiota in NTM-PD. By considering insights across anatomical sites, this review aims to contextualise the microbiome within multiple dimensions of NTM-PD, including host susceptibility, disease progression, treatment responsiveness, and the effects of antibiotic therapy. A better understanding of the microbiome in NTM-PD could hold promise in uncovering the complex and multifactorial mechanisms that contribute to the heterogenous clinical course and challenging management of NTM-PD.

## Section I: Background

### What is nontuberculous mycobacterial pulmonary disease, who does it affect and how is it treated?

**Nontuberculous mycobacteria (NTM)** are aerobic, non-motile opportunistic pathogens. NTM comprises a phylogenetically diverse group of environmental rapid- and slow-growing species and subspecies within the genus *Mycobacterium (M.)*, distinct from the *M. tuberculosis* complex and *M. leprae* [1]. Extrapulmonary NTM infections such as skin, soft-tissue and musculoskeletal infections occur, but pulmonary infection and disease predominates clinically [2]. Laboratory isolation of NTM can reflect transient infection and is not always indicative of disease, making it challenging to make the distinction. Guidelines for the diagnosis of NTM-PD (rather than infection) require clinical, radiographic and microbiological criteria all be met [2,3]. The significance of NTM-PD is increasing globally, as rates of NTM pulmonary isolation and disease continue to rise [4]. *M. avium complex* (MAC), slow-growing NTM, and *M. abscessus*, rapid-growing NTM, are the most common causative agents in NTM-PD [5–7], both contributing substantially to morbidity and mortality [8,9].

Clinically, NTM-PD is commonly broadly categorised into two distinct clinical phenotypes [3,10]. Fibrocavitary disease involves inflammatory cavitation, progressive scarring and surrounding fibrosis in the lung. It is often seen in middle-aged men with a history of smoking and excessive alcohol consumption, and underlying lung diseases such as past tuberculosis, chronic obstructive pulmonary disease (COPD) and emphysema [3]. Nodular bronchiectatic NTM-PD is commonly associated with the ‘Lady Windermere’ phenotype [11], characterised by distinct host morphological traits; typically peri- or post-menopausal women, with above average height and lean body habitus, high rates of thoracic deformities (scoliosis and pectus excavatum) and mitral valve prolapse, despite the absence of a shared immune or genetic profile [12–14].

Current NTM-PD guideline therapy entails prolonged multidrug regimens and is unacceptably long, usually at least 12–24 months [2]. The causative NTM species, mycobacterial susceptibility to available antibiotics, disease phenotype, patient characteristics and drug tolerability are just some of the many factors that influence the choice and success of NTM-PD therapy [2,15]. Clinical experience indicates that adverse effects from NTM-PD treatment are common, occurring in nearly 40% of patients, irrespective of NTM species [16]. Treatment success, referring to clinical cure, is low at only 40–60% [17,18]. Furthermore, rates of microbiological recurrence and chronically progressive NTM-PD are high, and often represent reinfection rather than relapse [18–20]. Given the substantial treatment burden, high incidence of adverse effects, and limited long-term efficacy, there is an urgent need to optimise our understanding of NTM-PD.

**Fig 1:**
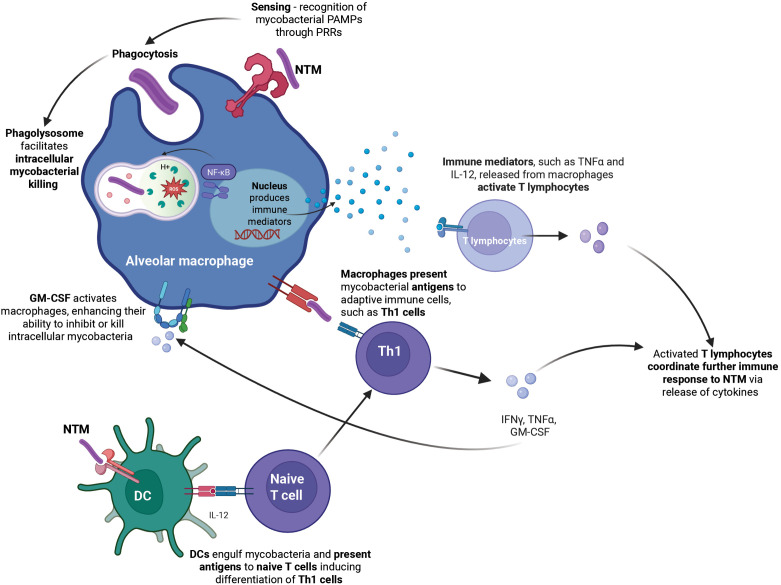
Innate and adaptive immune response to NTM infection in the lung. Alveolar macrophages are integral in the initiation of an immune response against inhaled NTM. This occurs in part through phagocytosis of NTM as well as the release of chemokines and cytokines by alveolar macrophages that recruit additional macrophages and other phagocytes to the site of infection. Macrophage activation by NTM is facilitated by pattern recognition receptors (PRRs), of which Toll-like receptor 2 (TLR2) plays a central role [28–31]. Macrophage recognition of NTM initiates phagosome maturation, lysosome acidification and antimicrobial defence mechanisms (e.g., generation of reactive oxygen species, expression of degradative enzymes), facilitating intracellular killing of mycobacteria [32]. Dendritic cells loaded with mycobacterial antigen that migrate to draining lymph nodes present mycobacterial antigens to naïve T cells, thereby facilitating T cell differentiation [33]. The associated cytokine milieu, such as via interleukin (IL)-12 release, shapes T cell differentiation into T helper 1 (Th1) cells. Infected macrophages also present mycobacterial antigens to Th1 cells. Th1 cells, alongside other innate and adaptive lymphocytes, coordinate mycobacteria-restricting immune responses, including interferon (IFN)-γ, tumour necrosing factor (TNF-α), and granulocyte macrophage colony-stimulating factor (GM-CSF)-mediated macrophage activation [34–37]. Fig 1 created with Biorender.com.

In healthy individuals, NTM are eliminated through coordinated innate and adaptive immune responses (Fig 1). Prior reviews have highlighted the complexity of these NTM–host immune-interactions [21–25]. However, it must be noted that current understanding of these interactions is commonly derived from models that do not reflect the specific host environments encountered in NTM-PD (such as *Drosophila melanogaster* [26] or Zebrafish embryo [27]), with knowledge gaps often filled by extrapolation from the more extensive understanding of host defence mechanisms against *M. tuberculosis*.

Susceptibility to NTM infection and NTM-PD is associated with a spectrum of immune dysfunction, ranging from overt primary (e.g., chronic granulomatous disease) or acquired immunodeficiency (e.g., chronic immunosuppressive drug exposure) [14,38–40]. However, in immunocompetent hosts, including individuals with the ‘Lady Windermere’ phenotype or underlying chronic lung disease, no single genetic or immunological defect has been identified to account for NTM susceptibility. Instead, we hypothesise that such susceptibility may arise from subtle or combined impairments in immune cell functions. Indeed, persistent NTM infection and/or NTM-PD are associated with reduced absolute counts of adaptive immune cells [41,42], reduced cytokine production (e.g., interleukin (IL)-1, IL-10, IL-18) [43], and/or immune cell exhaustion, suggesting that broader immune dysregulation could underlie susceptibility [12,43–47]. Toll-like receptor 2 (TLR2) is a pattern recognition receptor central to host recognition of MAC [28,30], and *M. abscessus* [29,31]. In peripheral blood monocytes from NTM-PD patients following MAC stimulation, TLR2 mRNA expression was reduced and cells showed diminished IL-12 and tumour necrosis factor (TNF)-α production [48]. Whether similar findings occur with *M. abscessus* stimulation remains to be determined. Thus, host susceptibility to NTM-PD appears to be underpinned by subtle and/or combined immune impairments, and the extent and combinations might vary between individuals. Detailed characterisation of immune responses in individuals with known NTM-PD susceptibility, prior to and during NTM infection, is needed to delineate the precise mechanisms underlying susceptibility and to better categorise susceptibility phenotypes in this multifactorial disease.

An important factor in addressing these knowledge gaps is that different NTM species appear to have unique mechanisms to subvert host defence [49–51]. For example, MAC replicates and persists intracellularly [49], through disrupting intracellular vesicle trafficking and impairing phagosome maturation [50]. In the context of *M. abscessus* infection, smooth and rough morphological variants show differential interactions with the immune system [52], where increased virulence of a *M. abscessus* rough variant was associated with phagosomal escape in macrophages, thereby triggering Type I interferon expression, cell death, and cell-to-cell spread [51]. In a comparison study of MAC-PD and *M. abscessus*-PD, multiparametric flow cytometry analysis of peripheral blood mononuclear cells indicated NTM-species-specific immune checkpoint marker ‘fingerprints’ in the host response: MAC-PD was associated with high T cell immunoglobulin and mucin-domain-containing protein 3 (TIM-3) expression by T cells; *M. abscessus*-PD was associated with high cytotoxic T-lymphocyte associated protein 4 (CTLA4) expression by CD4^+^ T cells (especially T-regulatory cells) [44]. Thus, characterisation of NTM-PD-associated host susceptibility will require consideration of the NTM species, strains, and morphotype.

### The microbiome in health and disease

The microbiome is increasingly recognised as a dynamic and influential modulator of health and disease. Broadly, the term “microbiome” refers to the genomic composition and diversity of a microbial community (i.e., the microbiota) and how that might be affected by the physicochemical properties of its surrounding environment (biome) at the time of sampling [53]. The prevailing view is that the microbiota associated with an individual outnumbers the absolute count of human somatic and germ cells by a factor of ten [54]. Accordingly, the collective DNA content of the microbiota, known as the metagenome, is phylogenetically more variable and dynamic compared to the relatively stable human genome [55,56]. Microbiome composition is influenced by physicochemical factors (such as oxygen and moisture levels, pH, and nutritional ecology), host-derived factors (including immune responses and genetics), and environmental or lifestyle factors (birth-delivery mode, breast- or formula-feeding, dietary pattern, exercise and exposure to antibiotics/medication) [55,57,58]. As such, differences in the relative abundance of microbial species and community composition lead to dynamic and variable microbiotas at different anatomical sites, even among healthy individuals [59]. Measures of the taxonomic diversity and relative abundance of the microbiota recovered from different body sites are often reported; based principally on case-control and/or cohort studies [60,61]. Reductions in these measures are commonly referred to as a dysbiosis, which often, but not always, is associated with disease [61]. There may be patterns that characterise disease-associated dysbiosis with some shared microbiome changes across different diseases, whilst other changes may be disease-specific [60]. Some diseases are associated with the presence of pathogenic microbes in the microbiota whereas others are characterised by a relative reduction in microbes associated with health [60]. Indeed, although microbiome research generally suggests that susceptibility to and/or pathophysiology of various diseases may be associated with taxonomic variations in the microbiome, there is consensus that the concept of a ‘healthy’ microbiome lacks a universal definition and objective criteria [54,55,62].

### The lung microbiota: Could it be more stable in respiratory disease states than in health?

The lungs were historically believed to be sterile, but it is now established that the lungs host a resident microbiota distinct from the oropharynx [63,64]. Distal airways may have lower microbial richness and the right upper lobe is suggested to most closely resemble the oral cavity [64], therefore supporting the concept that the lung microbiota may be continually recolonised by microbes from adjacent sites and the environment through microaspiration and breathing [65,66]. Dickson and colleagues [67] propose that microbial composition in the airways is governed by a dynamic balance of three key factors; microbial immigration, microbial elimination and regional growth conditions. In the context of lung pathologies, it is hypothesised that alterations in the local airway environment, be it structural, immunological or metabolic, create conditions more conducive to bacterial proliferation, whereby microbial growth may surpass the airways’ capacity for elimination, this can result in persistent microbial colonisation and dysbiosis [67,68]. The relationship between the lung microbiota and respiratory diseases has been previously reviewed in the literature [69,70], and is relatively well-characterised in several chronic respiratory diseases that are associated with the development of NTM-PD. Shifts in microbial composition and the presence of certain taxa have been observed when comparing acute exacerbation states to clinically stable periods in COPD [71] and bronchiectasis [72]. These exacerbations are typically marked by reduced microbial diversity, associated with overgrowth of clinically significant pathogens such as *Pseudomonas (P.) aeruginosa* or *Haemophilus influenzae* [71]. A similar reduction in diversity is seen in cystic fibrosis patients chronically, but not intermittently, colonised by *P. aeruginosa* [73]. This pattern of dysbiosis, and the persistent dominance of certain taxa, may contribute to disease progression, supporting the hypothesis that the lung microbiota in respiratory disease is more stable and enduring than in health [65].

### At the intersection of the lung microbiota and host susceptibility in NTM-PD

**Fig 2:**
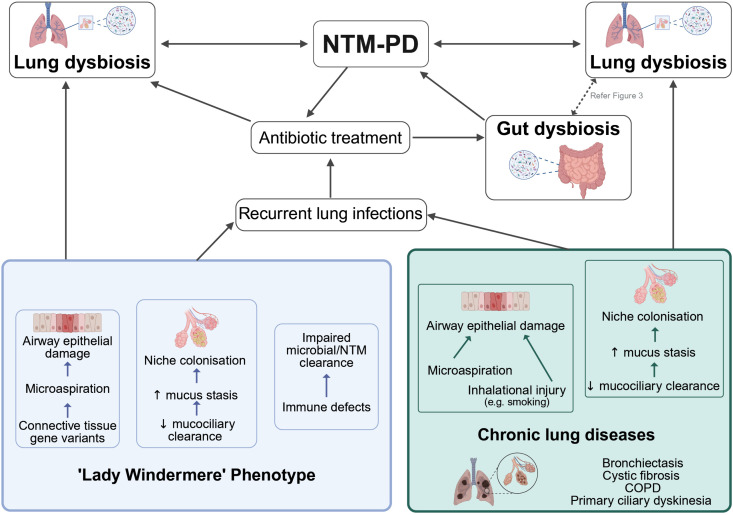
Model of host susceptibility factors driving lung and gut dysbiosis in NTM-PD. The model is divided into two key categories of susceptible individuals: the ‘Lady Windermere’ phenotype and chronic lung disease. These categories are not mutually exclusive, and some individuals may exhibit features of both (or in fact neither). Common to both lies airway damage, impaired mucociliary clearance, and mucus stasis, which may promote niche colonisation by NTM or other pathogens, leading to recurrent infections and often requiring antibiotic use. This, in turn, may cause further dysbiosis in the lung and perturb the gut microbiota (see Fig 3). Fig 2 created with Biorender.com.

While environmental factors (e.g., temperature, rainfall) may influence the nature and degree of exposure to NTM [5], most individuals are able to control and eliminate NTM upon exposure. In cases where NTM infection can occur and progress to NTM-PD, underlying host susceptibility is multifactorial and multigenic [74,75]. At present, linking specific immune susceptibility factors to the microbiota in NTM-PD remains challenging, as we do not understand the varying combinations of subtle immune defects that may underlie susceptibility in different individuals. Broadly; however, immune dysfunction may contribute to pulmonary dysbiosis by impairing microbial clearance, allowing microbial expansion and promoting disease pathogenesis. In the following section, we examine the potential interplay between the lung microbiota, and two key susceptibility factors, impaired mucociliary clearance and microaspiration (see Fig 2), which are thought to predispose NTM infection and disease, and are also implicated in several chronic pulmonary conditions commonly associated with NTM-PD.

#### Mucociliary dysfunction and mucus stasis: Creating niches for lung dysbiosis in NTM-PD.

The mucosal interface [76] and mucociliary clearance play a crucial role in airway immune defence, as ciliary motion coordinates transport of microorganisms and inhaled particles out of the respiratory tract [77]. Bronchiectasis [78,79], COPD [80,81], and primary ciliary dyskinesia [79,82], are all diseases associated with both impaired mucociliary clearance, and increased risk of NTM colonisation, infection and disease. NTM infection is also common in individuals living with cystic fibrosis [83], and even without overt cystic fibrosis disease, cystic fibrosis transmembrane receptor (CFTR) mutations are frequently observed in individuals with NTM-PD [13,84]. In the absence of concurrent underlying lung conditions, primary respiratory epithelial cells isolated from individuals with NTM-PD (including MAC and *M. abscessus*) showed abnormally low resting ciliary beat function compared to healthy controls [85]. Additionally, transcriptomic analysis of normal primary human bronchial epithelial cells infected with either MAC or *M. abscessus* revealed downregulation of genes associated with ciliary function. This suggests a potential mechanism by which NTM infection itself may impair mucociliary clearance in the respiratory epithelium, in the absence of other known risk factors that enhance susceptibility to NTM-PD [86]. Thus, in the presence or absence of pulmonary co-morbidities, impaired mucociliary clearance is a common susceptibility factor that may underlie NTM-PD.

Impaired mucociliary clearance and increased mucus production in respiratory disease can result in mucus stasis and hyper-concentrated mucus within the airways [87–89]. Mucus stasis can create a localised niche with altered environmental conditions, such as hypoxic gradients [90] and changes in nutrient availability [91], which may alter host–pathogen interactions [92], and propagate bacterial colonisation [93,94]. The precise nature and dynamics of these localised niches, including how different environmental, host, and disease factors interact still needs to be investigated, and will likely vary between diseases, so should be considered in NTM-PD. We hypothesise a similar mechanism within cavities in NTM-PD, where the cavity could serve as a localised niche that allows bacterial proliferation. One recent study found that disease-involved sites in the fibrocavitary form of NTM-PD harboured more dominant bacterial genera and a higher proportion of *Mycobacterium* compared to the nodular bronchiectatic NTM-PD group [95]. These findings cautiously suggest that the mycobacterial burden may be higher in the fibrocavitary form, however, further research is needed to understand the dynamics of microbial communities within NTM-PD cavities. Research on mucociliary clearance and mucus stasis has thus far primarily focussed on cystic fibrosis, and indeed, the recent successful introduction of CFTR modulators that confer a significant improvement in mucociliary clearance [96] are likely, at least in part, responsible for the reduction of lung infections by key cystic fibrosis pathogens [97], including NTM infections [98]. We propose that in NTM-PD, impaired mucociliary clearance affects the microbiome’s composition and this enables susceptibility to colonisation by NTM. This colonisation by NTM species could then drive further lung microbiota dysbiosis and persistent inflammation. The interaction between lung dysbiosis, mucociliary clearance and NTM-PD warrants further study. 

#### Microaspiration silently driving lung dysbiosis in NTM-PD.

Microaspiration occurs due to the contiguous nature of the oropharyngeal and tracheobronchial tree and represents a direct connection between the microbial communities in the gut and lung. Longitudinal analysis of paired gut and lung microbiota samples in infants has shown that several bacterial taxa appear in the gut prior to detection in the lung, supporting the concept of a direct microbial connection between these sites [99]. Even in health, microaspiration-mediated enrichment of oral commensals in the lung microbiota has been shown to modulate pulmonary cellular immune responses, with evidence of a pro-inflammatory phenotype reflected by upregulation of inflammatory cytokines [100]. Microaspiration may be more prevalent in individuals with chronic lung diseases. In an observational study investigating the presence of gastroesophageal reflux disease, increased oesophageal acid exposure was observed in 37% of COPD patients, 40% of bronchiectasis patients and only 18% of controls [101]. In NTM-PD (MAC-PD and *M. abscessus*-PD), increased oesophageal acid exposure was reported in 26% of a nodular bronchiectatic cohort [102] and another study reported micro-aspiration in 87% of NTM-PD patients [103]. Whilst microaspiration often remains clinically silent (e.g., 70% of individuals in a cohort with nodular bronchiectatic disease [102]), we hypothesise it is nonetheless relevant and may contribute to lung dysbiosis through multiple mechanisms. Firstly, increased microaspiration associated with disease states may enhance the influx of non-resident microbial taxa into the lower respiratory tract, disrupting the equilibrium between microbial immigration and elimination. Such disturbances are likely to alter local ecological conditions and may promote colonisation by opportunistic species. For example, in a cohort of 47 individuals clinically suspected of having NTM-PD, those with reflux exhibited significantly reduced diversity of the microbiota in bronchoalveolar lavage fluid (BALF), driven by predominance of select taxa such as *P. aeruginosa,* compared to the non-reflux group [104]. Secondly, microaspiration may be linked to impaired mucociliary clearance, as microaspiration models of cultured human primary bronchial epithelial cells show cell damage and death when exposed to bile acids [105]. Additionally, bile acid may directly influence microbial behaviour in the lung. For example, exposure to bile acids has been shown to induce biofilm formation in *P. aeruginosa* isolated from cystic fibrosis patients *in vitro* [106]. *In vivo*, bile acid aspiration in people with cystic fibrosis was associated with increased colonisation by *P. aeruginosa* and upregulation of genes linked to chronic biofilm formation in sputum microbiota [107]. This biofilm formation was also linked to increased antibiotic tolerance, including macrolides. Therefore, microaspiration may contribute to dysbiosis in lung diseases such as NTM-PD and also interfere with our ability to treat disease, either directly by facilitating microbial immigration into the airway, or indirectly by inducing airway damage that could impair mucociliary clearance.

We postulate that microaspiration in NTM-PD may be associated with connective tissue-related gene variants, which were identified in 90% of individuals in a cohort of 69 NTM-PD patients [75]. Ninety-nine specific genes involved in the structure and regulation of connective tissue were included in this study. The affected genes span several categories critical for connective tissue integrity and function, including collagen genes (e.g., *COLA1*), proteoglycan genes (e.g., *ACAN*), matrix and regulatory genes (e.g., *ADAMTS2* for collagen processing), and signalling pathway genes involved in connective tissue maintenance and repair (e.g., *TGFB2*). Although this cohort was not stratified by radiological phenotype, 72% exhibited connective tissue features (scoliosis, mitral valve prolapse, pectus excavatum and/or joint hypermobility), suggesting that a substantial proportion may belong to the ‘Lady Windermere’ phenotype. This phenotype, as discussed, is characterised by ‘Marfanoid’ features and overlaps with connective tissue disease traits [12–14]. Notably, nodular bronchiectasis appears to be relatively common in individuals with Marfan syndrome [108], and both bronchiectasis and pulmonary NTM infections have been reported in those with Marfan and other heritable connective tissue disorders [109]. These observations suggest a possible link between connective tissue disease abnormalities or traits, oesophageal dysmotility and increased microaspiration, which may be particularly relevant to the nodular bronchiectatic or ‘Lady Windermere’ phenotype. Further studies are needed to clarify the functional impact of these variants and their role in disease.

### The gut–lung axis

**Fig 3:**
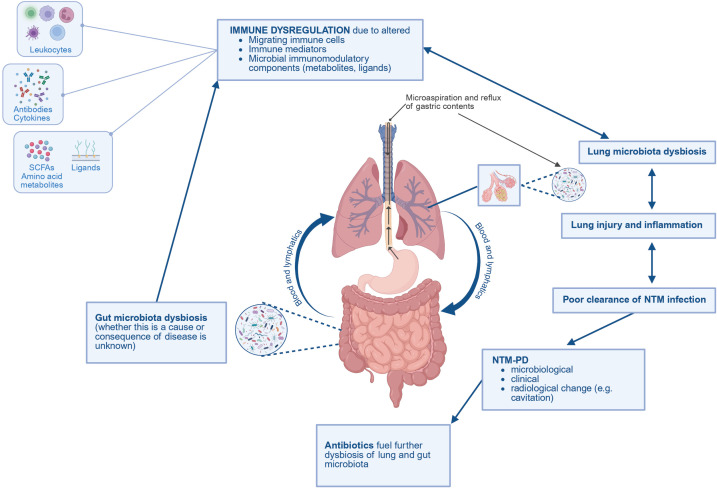
A working model of the gut-lung axis (GLA) in nontuberculous mycobacterial pulmonary disease (NTM-PD). The gut and lung have specialised microbiotas that are interconnected via the bloodstream and lymphatic system, via the GLA [65,68]. We propose gut microbiota dysbiosis as a potential initiating factor in the cycle of NTM-PD; however, whether it represents a cause or consequence of disease remains unclear. Dysbiosis contributes to changes in migrating immune cells, microbial immunomodulatory components and immune mediators, together representing a level of systemic immune dysregulation that affects the lung microbiota via the GLA [110–112]. Microaspiration and reflux are also implicated in the GLA and respiratory disease pathophysiology with a degree of direct seeding of gut microbes into the airways [99,104]. Lung microbiota dysbiosis may then cause lung injury and inflammation, contributing to poor clearance of NTM infection, allowing establishment of disease. Antibiotic treatment for NTM-PD then causes further dysbiosis of both the gut and lung microbiotas [61,113,114], so the cycle continues. Fig 3 created with Biorender.com.

Beyond direct anatomical connections such as in microaspiration, spatially distinct microbial communities do not operate as isolated populations. The lung environment and its associated microbiota are influenced by, and involved in complex interactions, including via the gut-lung axis. The gut-lung axis (GLA) describes the concept of the complex bidirectional microbiome–host interactions at each anatomical site, the gut and lungs, and the connectedness between these sites [65,66,68]. Microbial structural components and metabolic outputs from microbes possess immunomodulatory properties and can disperse via the bloodstream and lymphatic circulation, facilitating inter-niche communication that is fundamental in maintaining homeostasis and regulating host immune responses [110–112]. Fig 3 summarises the proposed roles of GLA in NTM-PD.

The gut microbiome, particularly within the distal (ileocolonic) regions of the gastrointestinal tract, has been extensively reviewed for its contributions to the development and regulation of peripheral immune functions, including in the lung [65,115,116]. Emerging evidence suggests that while the gut microbiota can be protective against respiratory infections [112], gut dysbiosis can impair immune tolerance and contribute to inflammation. Gut dysbiosis has been associated with manifestations and exacerbations in various respiratory disease contexts [117,118]. For example, in a murine model, reduced gut microbial diversity increased mortality from respiratory viral infection by disrupting regulatory T cell balance and elevating pro-inflammatory cytokines [119]. In COPD, faecal microbiota profiles were characterised by a *Prevotella*-dominated enterotype with reduced levels of short-chain fatty acids (SCFAs); faecal microbiota transplantation from these patients into mice induced ‘COPD-like’ lung inflammation and accelerated disease progression [120]. SCFAs such as acetate, propionate and butyrate, are produced in the gut as by-products of bacterial anaerobic fermentation of dietary fibre [121]. Their diverse physiological roles and implications for human health and disease were recently reviewed by Mukhopadhya and colleagues [122], including their regulation of local gut immune responses and their systemic immunomodulatory effects [110,111]. Altered SCFA metabolism has been identified in pulmonary diseases, with reduced abundance of SCFA-producing bacteria and/or decreased production of SCFAs reported in patients with bronchiectasis [123], active tuberculosis (TB) [124], and COPD [125]. To our knowledge, assessments on SCFA-focussed analyses in NTM-PD have not yet been reported. Such analyses, paired with immune profiling in gut and lung tissue microenvironments, would be highly valuable in determining whether altered SCFA metabolism contributes to NTM-PD and how it may influence the immune response to NTM.

One metabolite which has been examined in the context of NTM-PD is L-arginine, an amino acid obtained through dietary intake, but also synthesised endogenously, as well as produced by gut microbes. L-arginine is proposed to have roles within multiple metabolic pathways and to shape immune functions [126–128]. Kim and colleagues [129] reported decreased L-arginine in serum of *M. abscessus*-PD patients as well as in *M. abscessus*-infected mice. Oral administration of L-arginine to mice infected with *M. abscessus* was associated with remodelling of the gut microbiota, and a reduction in pulmonary NTM burden and amelioration of lung histopathology. Transcriptomic profiling of the lung tissues revealed elevated expression of interferon (IFN)-γ and IL-12 in these *M. abscessus-*infected mice treated with L-arginine, suggesting enhanced Th1-mediated pulmonary immune responses. This example highlights how gut microbial alterations via microbe-derived metabolites might influence immune responses and disease severity in the lungs. However, further research is needed to establish whether there is a causal relationship between L-arginine-induced changes in the microbiota and enhanced pulmonary defence, especially as current evidence is derived from murine-infected mice and may not fully translate to humans. Additionally, L-arginine may also exert effects through other mechanisms, such as increasing nitric oxide synthase activity [130], which is known to have antimicrobial properties against mycobacteria [131,132]. To define the specific roles of microbe-derived metabolites and better understand the mechanisms contributing to the GLA in NTM-PD, more detailed characterisation of the microbiota and their functional capabilities in this condition is required.

## Section II: Insights into the lung and gut microbiotas from studies in NTM-PD

### Challenges in the lung microbiota in NTM-PD: the need for disease stratification and methodological consistency

Variations in the lung microbiota are inconsistent across studies examining NTM infection and NTM-PD. Of note, there has been a transition in the field from culture-dependent to culture-independent methods of microbiota analysis. As culture-independent methods of surveying the microbiota are less affected by the biases introduced by culturing, we focussed our assessment on studies based on culture-independent analyses (details of studies outlined in Table 1).

**Table 1:** Summary table of culture-independent studies characterising the impact of NTM infection or NTM-PD on the lung microbiota.

Study population	Sample and sequencing	Significant findings	Ref
Bronchiectasis with respiratory symptoms and imaging abnormalities suggestive of NTM-PD (*n* = 106)	Sputum (*n* = 297), oral wash (*n* = 297) and BALF (*n* = 20); 16S rRNA sequencing	NTM^−^ and NTM^+^ sputum α- and β-diversity measures not significantly different. Sputum microbial composition more closely resembled oral wash microbiota than BALF. Sputum bacterial load ~log_2_ higher than BALF samples (but not reported if BALF corrected for dilution). β-diversity (UniFrac distance) measures significantly different between sputum and BALF samples. Enrichment of oxalobacteraceae (family) in NTM^+^ BALF samples. *NTM species: 92% MAC, 8% M. abscessus.* *Note: cohort was bronchiectasis patients suspected of having NTM-PD, prevalence of NTM*^*+ *^*was 58%, unclear whether NTM*^*+*^ *cohort represents NTM-PD or NTM-infection.*	[133]
NTM-PD (*n* = 14) vs. controls (*n* = 10)	PSB (*n* = 21), bronchial washing (*n* = 21); 16S rRNA sequencing	α-diversity (OTU and Chao1) were lower in NTM-PD than controls (but not statistically significant). Significantly lower β-diversity (weighted UniFrac distance) of PSB and bronchial washings in NTM-PD group. More genus *Pseudomonas* and *Rhodococcus* in NTM-PD. Microbiome composition similar in PSB and bronchial washing samples. *NTM species: 54.5% M. avium, 27.3% M. intracellulare, 9.1% M. kansasii, 9.1% unidentified.*	[139]
Stable NTM-PD (*n* = 21 baseline, *n* = 13 follow-up) vs. progressive NTM-PD (pre-antibiotic, *n* = 14)	Sputum (*n* = 48); 16S rRNA sequencing	Sputum α- and β-diversity measures not significantly different between the stable and progressive (to treatment) NTM-PD groups. *Porphyromonas pasteri*, *Haemophilus parahaemolyticus*, *Prevotella nanceiensis*, and *Gemella haemolysans*, associated with disease stability. Stable group: less genus *Bergeyella* and species *Prevotella oris* in spontaneous converters (*n* = 9) vs. persistent NTM-positivity (*n* = 12). More baseline genus *Haemophilus* and *Rothia* in treatment-responders. *Atopobium* and *Parvimonas* increased in treatment-refractory group. *NTM species: 66% MAC, 14% M. abscessus, 9% M. massiliense, 11% mixed infection.*	[135]
Bronchiectasis patients; NTM-positive (*n* = 29) vs. NTM-negative (*n* = 29) (based on BALF culture)	BALF from worst-infected lesion (*n* = 58); 16S rRNA sequencing	Diversity measures not reported. Anaerobes (*Prevotella*, *Fusobacterium*, *Propionibacterium* and *Veillonella*) significantly more abundant in NTM^+^ group. In NTM^+^ group, there was a significant relationship between scores for collapse/consolidation on high-resolution computed tomography and the ratio of *Prevotella* genus phylotypes (but not in the NTM^−^ group). *NTM species: 45% M. avium, 35% M. intracellulare, 14% M. kansasii, 3% M. abscessus, 3% M. chelonae.*	[145]
Controls (*n* = 5), NTM-PD (*n* = 5) and NTM-PD-Bca (breast cancer history, *n* = 15)	Sputum (*n* = 25); 16S rRNA sequencing	α-diversity (OTU and Shannon index) significantly higher in NTM-PD than controls. Significantly higher OTU in NTM-PD-Bca compared to controls. β-diversity (UniFrac distances) significantly different in controls versus NTM-PD-Bca. *Fusobacterium* enriched in NTM-PD. *NTM species: all NTM-PD was MAC-PD.* *Small numbers, pilot study with unclear cancer influence.*	[141]
Paired samples; NTM-PD diseased vs. non-diseased (*n* = 23)	Lung tissue specimens (from surgical lung resection, *n* = 46); 16S rRNA sequencing	Diseased sites had greater α-diversity (ACE, Chao1, Jackknife, Shannon index). Read counts generally higher at disease-involved sites, but not significantly. Differential proportions of microbial taxa in diseased vs. non-diseased sites: Non-diseased: significantly greater *Acinetobacter* and *Enhydrobacter.* Diseased: significantly greater Limnohabitans, Rahnella, Lachnospira, Flavobacterium, Megamonas, Gaiella, Subdoligranulum, Rheinheimera, Dorea, Collinsella and Phascolarctobacterium. Differential taxa enrichment based on causative NTM species (MAC or *M. abscessus*) Radiological phenotype comparison: Diseased: *Mycobacterium, Bacteroides*, *Faecalibacterium*, *Blautia* genus were more abundant in the fibrocavitary than nodular bronchiectatic NTM-PD, with significant β-diversity (PERMANOVA and UniFrac distances) differences. Non-diseased: *Escherichia* and *Streptococcus* were more abundant in fibrocavitary, with no diversity differences. *NTM species: 70% MAC-PD, 30% M. abscessus-PD.*	[95]
Bronchiectasis & suspected NTM infection (*n* = 38)	BALF (*n* = 69) from suspected NTM-infected region; Amplicon sequencing	18 NTM^+^ BALF samples: significantly lower α-diversity (Shannon diversity), higher relative abundance of *Mycobacterium*, *Pseudomonas*, *Veillonella*, *Actinomyces*, and *Fusobacterium*, and significantly lower *Staphylococcus*, *Haemophilus* and *Porphyromonas*. *NTM species: 50% M. avium, 28% M. intracellulare, 11% M. abscessus, 6% M. lentiflavum, 6% M. xenopi.*	[138]
Treatment-naïve females with NTM-PD (*n* = 10), vs. healthy females (*n* = 10)	Sputum (*n* = 10); 16S rRNA sequencing	Significantly lower α-diversity (ASV) and β-diversity (UniFrac distances) in NTM-PD. Genus *Sneathia* higher in NTM-PD and other species significantly different between groups (*Prevotella fusca*, *Prevotella marshii*, *Prevotella nanceiensis*, *Capnocytophaga ochracea*, *Gemella morbillorum*, *Streptococcus constellatus*, *Sneathia sanguinegens*, and *Treponema bryantii* dominant in the NTM-PD group compared to healthy controls. *NTM species: M. avium (40%), M. abscessus (20%) M. intracellulare (20%), M. massiliense (10%), mixed (10%).* *Note: faecal microbiota also studied, details found below in* Table 2.	[137]
MAC-PD (*n* = 9) vs. non-MAC-PD (*n* = 16) (based on BALF culture)	BALF (*n* = 25); 16S rRNA sequencing	No significant difference in diversity between two groups. Genus *Pseudomonas* absent in MAC+ samples, suggesting mutual exclusivity. *NTM species: all MAC-PD.* *Note: focus of the study was to compare detection methods; 16S sequencing was less sensitive for mycobacterial culture.*	[134]
Bronchiectasis with NTM-PD (*n* = 9) or without (*n* = 11) (based on BALF culture)	BALF samples; 16S rRNA sequencing	Greater α-diversity (Simpson index) in NTM-PD. Significant β-diversity (Bray-Curtis and weighted UniFrac distances) differences. More *Streptococcus*, *Prevotella*, and *Staphylococcus* in NTM-PD. More *Pseudomonas, Haemophilus,* and *Staphylococcus* in bronchiectasis group (without NTM-PD). *NTM species: 37.5% M. avium, 50% M. intracellulare, 12.5% M. mantenii.* *Note: did not stratify for disease severity or bronchiectasis exacerbation.*	[142]
NTM-PD (*n* = 126) with progressive (*n* = 49), or nonprogressive disease (*n* = 77)	Longitudinal sputum (follow-up 2 years or to disease progression); 16S rRNA	Progressive disease was associated with reduced α-diversity (Shannon and Simpson index). Significant β-diversity (PCoA and weighted UniFrac) differences between groups. Seven genera (*Burkholderia, Pseudomonas, Sphingomonas, Candidatus, Saccharibacteria, Phocaeicola, Pelomonas* and *Phascolarctobacterium*) were used to construct a prediction model for disease progression (high accuracy, area under curve = 0.871). *NTM species: 46% MAC, 33.3% M. abscessus, 20.6% M. kansasii.*	[140]
*M. abscessus* -PD; treatment-responder (*n* = 15) vs. non-responder (*n* = 12) (stratified on sputum mycobacterial culture a 2 weeks)	Sputum samples at baseline (pre-treatment), 2 weeks and 6 months into antibiotic treatment; 16S rRNA sequencing	α-diversity indices not different between the groups at each time point. Antibiotic treatment (azithromycin + amikacin + imipenem/cefoxitin ± clofazimine in 25 of 27 patients) caused significant perturbation of sputum microbiota. Longitudinal changes in serially collected sputum samples: Significant decreases in α-diversity (observed richness and Shannon index) in treatment-responder group whereas α-diversity remained consistent across time points in non-responders. β-diversity: significant change from baseline to 2 weeks in responders (not in non-responders), but both groups showed a significant change from baseline at 6 months. Responders show earlier changes in sputum microbiota due to antibiotic treatment than non-responders. Baseline increase in *Burkholderia Caballeronia, Paraburkholderia* and *Porphyromonas* abundance and 2-week decreased *Rothia* abundance were associated with treatment response. NTM subspecies (*massiliense* versus *abscessus*), presence of cavities and use of probiotics were all found to significantly alter some diversity and taxonomy measures in sputum. *NTM species: all M. abscessus, 48.1% subspecies abscessus.* *Note: faecal microbiota also studied, details found below in* Table 2.	[136]
Individuals clinically suspected of having NTM-PD (*n* = 47), with (*n* = 22) and without reflux (*n* = 25)	BAL, 16S rRNA sequencing	Presence of reflux linked to reduced α-diversity (Shannon diversity). Predominance of *P. aeruginosa* in the reflux group, compared to the non-reflux group. *NTM species: unspecified.* *Note: this study more focussed on the possible role of reflux rather than microbiota composition because of disease.*	[104]
Cystic fibrosis patients with transient NTM-infection or NTM-PD (*n* = 24)	Time-series analyses of sputum samples (*n* = 188), 16S rRNA sequencing	Specific diversity measures not reported, study focussed on microbial network structure to infer community stability and connectedness. Increasing relative abundance of certain taxa (*Pseudomonas*, *Streptococcus*, *Veillonella*, *Prevotella*, and *Rothia*) associated with subsequent diagnosis of NTM-PD and with persistent NTM infection (time-series analyses of sputum samples). Differences in network clustering between persistent and transient NTM infections; microbial network clustering more densely interconnected and less modular microbial community structure. *NTM species: transient NTM-infection—56% MAC, 25% M. abscessus, 19% Other; NTM-PD – 75% MAC-PD, 25% M. abscessus-PD.*	[143]

Abbreviations: BALF, bronchoalveolar lavage fluid; PSB, protected specimen brushings; OTU, operational taxonomic unit; ACE, abundance-based coverage estimate; ASV, amplicon sequence variant; PCoA, principal coordinate analysis.

To date, there is no clear consensus on whether NTM infection or NTM-PD is consistently associated with changes in microbial diversity. Some studies report no change in diversity [133–136], others observe a decrease [137–140], and few report an increase [95,141,142]. One hypothesis is that microbial diversity remains stable during transient infection, but declines with disease progression. This pattern mirrors observations in chronic *P. aeruginosa* infection in cystic fibrosis, where diversity declines only once chronic infection is established [73]. However, testing this hypothesis in NTM-PD is challenging due to the difficulty in distinguishing transient NTM infection from true NTM-PD in some individuals. Sulaiman and colleagues [133] found no difference in diversity between NTM-positive and NTM-negative bronchiectasis samples. However, all participants in this study exhibited respiratory symptoms and imaging abnormalities suggestive of NTM-PD, making it difficult to determine whether they were transiently infected or had established disease. Alternatively, the similar diversity observed between groups could reflect a shared underlying lung pathology, namely bronchiectasis. No difference in diversity was also observed in a study by Song and colleagues [135], which compared stable NTM-PD and progressive NTM-PD cases (those requiring treatment). This similarity could reflect that both groups had established disease, and that difference might only emerge when compared to transient infection—a group not included in the study. Future research would benefit from longitudinal studies that can track changes in the lung microbiota from transient infection through to established NTM-PD and progressive NTM-PD. One such study involving cystic fibrosis patients with NTM infection or NTM-PD, although not reporting specific diversity metrics, identified distinct differences in microbial network clustering between transient NTM infection and persistent NTM-PD [143]. The networks suggested that microbial communities in persistent NTM-PD were more interconnected and less modular, indicating reduced community stability in disease. Although not all individuals with NTM-PD follow a trajectory from transient infection to progressive disease, such analyses may offer valuable insights into the relationship between lung microbiota dynamics and disease progression.

In studies reporting decreased diversity, overgrowth of specific taxa is often observed. For instance, in a bronchiectasis cohort, NTM-positive individuals exhibited significantly reduced diversity compared to NTM-negative counterparts, with overrepresentation of *Mycobacterium*, *Pseudomonas*, *Veillonella*, *Actinomyces*, and *Fusobacterium* [138]. Similarly, decreased diversity in an NTM-PD cohort compared to controls was observed alongside overgrowth of *Pseudomonas* and *Rhodococcus* [139]. These findings align with observations in other chronic lung diseases, where reduced microbial diversity is seen in established disease and often driven by the dominance of specific taxa [71,73,144]. Of the studies reporting increased microbial diversity associated with NTM-PD, one was a pilot study significantly limited by sample size [141], the other provides a paired analysis of disease-involved segments compared to non-involved segments in resected lung specimens from individuals with NTM-PD [95]. It is possible that in advanced NTM-PD (such as cases requiring surgical resection) microbial diversity increases as the disease progresses, potentially due to invasion by other bacterial species or immune exhaustion. To better understand these dynamics, further research is needed to quantify changes in microbial diversity across the spectrum of NTM-PD severity.

Specific microbial taxa have been proposed as potential biomarkers for distinct disease characteristics in NTM-PD; however, substantial variability between studies limits the identification of consistent markers and their clinical applicability. Nevertheless, some patterns may be beginning to emerge. Anaerobic genera such as *Veillonella* [137,138,143,145], *Fusobacterium* [138,145], *and Prevotella* [145] are frequently reported at higher relative abundance in individuals with NTM infection or NTM-PD compared to healthy controls or those with NTM-negative bronchiectasis. These same taxa are among the most common in health and other chronic respiratory diseases [63,146], suggesting their presence is not NTM-PD specific. Whether the increased abundance of these anaerobic microbes could relate to changes in the airway microenvironment in individuals with NTM-PD, such as the development of hypoxic niches driven by mucus stasis [90], could be a future research question. The potential for sputum microbiota biomarkers to distinguish between disease progression and stability in NTM-PD has also been suggested, but findings remain inconsistent and unvalidated. For instance, *Prevotella nanceiensis* was found in one study as a marker of NTM-PD stability [135], while another study constructed a predictive model for NTM-PD progression using seven genera (*Burkholderia, Pseudomonas, Sphingomonas, Candidatus, Saccharibacteria, Phocaeicola, Pelomonas* and *Phascolarctobacterium*) [140]. Among these genera, *Pseudomonas* is frequently reported, albeit inconsistently. *Pseudomonas* abundance was increased in a study in NTM infection [138] and a study in NTM-PD [139], yet another study found it absent in all MAC-PD samples, with authors proposing a possible mutual exclusivity between *Pseudomonas* and MAC in the lung environment [134]. While such biomarkers may hold promise for future disease monitoring, the variability across studies currently limits the development of robust predictive models or biomarkers for disease progression.

We purport that the variability in findings and absence of a distinct microbial profile compared to healthy lungs reflects methodological heterogeneity rather than the absence of a disease-associated microbiota in NTM-PD. Most studies have included varying proportions of different species within their NTM-PD cohorts [95,133,135,137–139,142,145], primarily MAC and *M. abscessus,* but also some with smaller percentages with less commonly seen pathogens in NTM-PD such as *M. xenopi* and *M. lentiflavum* (specific percentages for each study are detailed in Table 1). Few studies have focussed on a single species, specifically MAC-PD [134,141], or *M. abscessus* NTM-PD [136]. This lack of species-specific focus is problematic, as data suggests differential taxa enrichment in lung tissue microbiota depending on NTM causative species (MAC vs *M. abscessus*) [95], and even potential differences in sputum diversity and taxonomy based on subspecies of *M. abscessus* (*massiliense* versus *abscessus*) [136]. Future studies should, at a minimum, ensure species-level consistency within NTM-PD groups, with consideration given to subspecies-level distinctions as well. Similarly, there may be differences in microbiota composition associated with radiographic phenotype (fibrocavitary versus nodular bronchiectatic) [95]. However, most studies have neither stratified participants by phenotype nor consistently reported it. Incorporating radiographic phenotype into future analyses may help clarify microbiota-disease associations and improve the interpretability.

Inconsistencies in the types of respiratory samples used to assess the lung microbiota in NTM-PD significantly hinder cross-study comparisons. Sputum samples are widely used in clinical and research settings due to their ease of collection and capacity to capture a broad range of microbial genera present in the lungs. Comparative analyses have shown that bronchoscopy does not yield additional genera beyond those identified in sputum samples [147]. Nevertheless, sputum represents a surrogate measure of the lower respiratory tract, as it comprises a composite of mucus from the lower respiratory tract and aqueous secretions from the oral cavity acquired during transit through the upper airway. This passage introduces contamination from oropharyngeal sources [147,148], which can confound interpretations of microbial composition. Studies comparing sputum with BALF and protected specimen brushings [147], and comparing sputum with direct lung sampling acquired at the time of transplantation [148], both in cystic fibrosis patients, reported significant differences in both microbial diversity and relative abundance, underscoring the limitations of sputum as a proxy for the lung microbiota. Similarly, a comparison of diversity and bacterial load in a subset of bronchiectasis patients, both NTM-positive and NTM-negative, suggested sputum microbial composition more closely resembled that of the oral cavity than BALF samples [133]. Other sampling techniques may be less affected by contamination. Intraindividual inter-site analysis in healthy individuals suggests BALF is not confounded by contamination from the oral microbiota [63]. Protected sampling methods, particularly protected specimen brushings (PSB), have also been shown to reduce oral contamination, potentially yielding even greater distinction from oral microbiota than other bronchoscopic techniques [149]. In NTM-PD, there were discrepancies when comparing protected specimen brushing and bronchial washing samples, with lower diversity in protected specimen brushings [139]. The lung microbiota is likely more accurately characterised using bronchoscopic samples than sputum samples, however, the clinical risks, invasiveness, and time-consuming nature of bronchoscopy limit its routine use. Nevertheless, to characterise the lung microbiota in NTM-PD, future studies require greater methodological consistency to allow for better comparison across the literature.

### The gut microbiota in NTM-PD: many questions to be answered

Gut microbiota dysbiosis has been associated with pulmonary disorders, such as COPD [117], bronchiectasis [123], and cystic fibrosis [150], but specific studies of gut-associated dysbiosis in NTM-PD are limited. To date, three studies have reported on the faecal microbiota in NTM-PD patients [details in Table 2] [136,137,151].

**Table 2:** Summary table of studies characterising the faecal microbiota in NTM-PD.

Study population	Significant findings	Ref.
10 healthy females vs. 10 females with NTM-PD (9 treatment naïve, 1 treated >12 months ago)	α-diversity (Shannon and ASV richness index) significantly decreased in NTM-PD group compared to HC. β-diversity (UniFrac distances) significantly decreased in NTM-PD. Increased genus *Sneathia* in NTM-PD than HC and 8 species dominant in NTM-PD group (*Prevotella fusca*, *Prevotella marshii*, *Prevotella nanceiensis*, *Capnocytophaga* ochracea, *Gemella morbillorum*, *Streptococcus constellatus*, *Sneathia sanguinegens*, and *Treponema bryantii*). *NTM species: 40% *M. avium*, 20% *M. abscessus,* 20% *M. intracellulare*, 10% *M. massiliense*, 10% mixed infection.* *Limitations: no reporting of disease severity, small sample size with significant patient heterogeneity (e.g., NTM-PD group significantly older and lower BMI than HC). Mix of phenotype, but mostly nodular bronchiectatic phenotype (90%).*	[137]
19 NTM-PD patients vs. 25 HC	α-diversity (Shannon and Chao1) significantly lower in NTM-PD group. β-diversity (UniFrac distances and PCoA) significantly different between groups. 11 bacterial genera were significantly different between NTM-PD and HC. NTM-PD patients had: Significantly lower relative abundance of Prevotellaceae family, *Prevotella_9* genus, Selenomonadales order, Negativicutes class, Veillonellanceae family, and *Megmonas* genus. Significantly greater abundance of Bifidobacterium longum subspecies longum, Clostridium species, Eubacterium hallii, and Ruminococcaceae UCG_014 genus. *Prevotella_9* genus, particularly *P. copri,* was significantly negatively correlated with disease severity measures [cavity presence, sputum acid-fast smear grading and radiographic score]. *Clostridium innocuum* group was significantly positively correlated with disease. *M. kansasii* mouse model Mice were exposed to 3 weeks of antibiotic-treated drinking water (containing ampicillin, metronidazole, neomycin sulfate and vancomycin) then infected with *M. kansasii* two days after cessation of antibiotics. Mice showed gut dysbiosis, a reduction in TLR2 activation activity in faeces, sera and lung tissue. Transcriptomic analysis showed genes associated with impaired immunity in the intestine and lung tissues. Oral administration of *Prevotella copri* (or its capsular polysaccharides), to the mice was then linked to heightened TLR2 signalling and correlated with enhanced immune response and decreased NTM-PD susceptibility. *NTM species: 72% M. avium complex, 12% M. kansasii, 12% M. abscessus complex, 4% other.* *Note: sputum sample results displayed in* Table 1. *Limitations: NTM-PD group significantly older, more likely to be female and had lower BMI than HCs. Heterogeneity of NTM group, mix of nodular bronchiectatic and fibrocavitary phenotype, causative organisms and disease severity.*	[151]
*M. abscessus-*PD (*n* = 27 patients) categorised as treatment-responder (*n* = 15) and non-responder (*n* = 12) based on sputum mycobacterial culture results at 2 weeks of treatment.	Faecal samples taken at baseline (pre-treatment), 2 weeks and 6 months into antibiotic treatment. **α-diversity:** Observed richness at 6 months and Shannon index at 2 weeks and 6 months were significantly lower in responders than non-responders. No significant difference in α-diversity between groups at baseline. **β**-diversity:**** no significant difference between groups at any time point. Taxonomic differences between responder and non-responder groups: At baseline: no distinct differences in major genera (>5% relative abundance) but *Eubacterium hallii* determined as key feature for discriminating the two groups (using Random Forest and LEfSe analysis), with numerically higher relative abundance in non-responders compared to responders (did not reach significance, *p* = 0.054). At 2 weeks: increased *Enteroccocus* was linked with treatment responsiveness (Random Forest and LEfSe analysis). Impact of *M. abscessus* treatment on the microbiota: Regimen: 25 of 27 patients on regimen consisting of Azithromycin+Amikacin+Imipenem/Cefoxitin + /-Clofazimine (specific regimens determined by physician). Typically 2 weeks intensive intravenous antibiotic therapy. No significant differences in treatment regimen between responder and non-responder groups. α-diversity: significantly decreased from baseline at 2 weeks and 6 months in the responder group, no significant changes in non-responder group across time points. β-diversity: both responders and non-responders had significant shifts in microbial communities from baseline, especially by 2 weeks, with changes stabilising by 6 months. Taxonomic changes: *Firmicutes* relative abundance significantly decreased from 83.2% at baseline to 60.8% in non-responders, whereas levels were relatively stable in responders. Generalised linear mixed model showed changes in *Firmicutes* abundance was not significant between the two groups (*p* = 0.058). At 2 weeks, *Enterococcus* relative abundance had increased in both groups compared to baseline, with a more pronounced increase in responders than non-responders. *Enterococcus* also became the key taxon for distinguishing the two groups, with increased abundance at 2 weeks linked to treatment responsiveness. *NTM species: all *M. abscessus*-PD (48.1% subspecies *abscessus*).* *Note: sputum sample results displayed in* Table 1. *Limitations: treatment dose and duration not noted in results, analysis was not adjusted for macrolide susceptibility or cavity presence as covariates due to relatively small sample size. Authors acknowledge changes between baseline and 2 weeks may also reflect changes in diet due to hospitalisation during this period.*	[136]

All studies utilised 16S rRNA (V3-V4 region) of faecal samples.

Abbreviations: HC, healthy controls; ASV, amplicon sequence variant; BMI, body mass index; TLR2, Toll-like receptor 2; LEfSe, linear discriminant analysis effect size.

Gut microbiota profiles may differ between individuals with NTM-PD (who are treatment naïve) when compared to healthy controls. Choi and colleagues [137] and Lin and colleagues [151] reported significantly decreased diversity measures in faecal microbial profiles of individuals with NTM-PD compared to healthy controls. Both studies included relatively small sample numbers and were unable to stratify by NTM-PD phenotype, causative NTM species, or disease severity. Broadly, the observed decrease in diversity in NTM-PD, corresponds with the decrease in diversity seen in other infectious [124,152] and non-infectious lung pathologies [123]. Changes in taxonomy; however, were not consistent across the two NTM-PD studies despite both using linear discriminant analysis effect size (LEfSe) to compare taxa abundances between groups and to determine statistically significant biomarkers [137,151]. Both studies identified that members of the genus *Prevotella* were altered in NTM-PD patients [137,151]. However, Choi and colleagues [137] reported that *Prevotella fusca*, *P. marshii*, and *P. nanceiensis* were predominant in NTM-PD subjects, whereas Lin and colleagues [151] saw reductions in the relative abundance of *Prevotella_9* in NTM-PD patients. Species of the genus *Prevotella* synthesise SCFAs [153], which might offer potential focus for future studies to integrate microbial taxonomy and their metabolic capabilities in NTM-PD. The variable findings related to *Prevotella* may arise from inconsistent definitions of a ’healthy’ control population, which are rarely reported in microbiota studies. We propose that the most appropriate matched control would be individuals with similar underlying host phenotype and lung disease, but without NTM infection, recent antibiotic exposure, or other significant co-morbidities that may be affecting the microbiome. Identifying such controls is challenging, so studies may benefit from multiple comparison groups. Greater confidence in characterising NTM-PD-associated gut dysbiosis at the genus or species level will also require larger patient and control cohorts, and more detailed consideration of clinical parameters including, but not limited to, NTM causative species, disease severity and concomitant diseases or infections.

Gut microbiota profiles may also be related to disease severity measures or disease exacerbation. Decreased gut microbiota diversity and richness has been seen in acute exacerbations of COPD compared to stable disease [144], and in acute exacerbation of bronchiectasis compared to stable bronchiectatic disease [123]. In NTM-PD, Lin and colleagues [151] found that 11 bacterial genera had significantly different abundance compared to a healthy control groups. These 11 genera were correlated with NTM-PD disease severity measures, including the presence of a cavity, grading of sputum acid-fast smear and radiographic scores [151]. The strongest correlation was observed between greater disease severity and reduced levels of the *Prevotella_9* genus [151]. *Prevotella* again emerges as a possible genus of interest, with potential relevance to NTM-PD severity. These findings warrant validation in larger, well-characterised NTM-PD cohorts. Further research should explore whether *Prevotella,* or other taxa, could serve as biomarkers of NTM-PD progression, and whether associated microbial shifts reflect changes in host metabolism, such as altered SCFA synthesis.

### Antibiotic treatment and the gut microbiota in NTM-PD

Antibiotic treatment causes significant disruption of the gut microbiota, affecting both composition and abundance to varying degrees [61,114]. Antibiotics have different modes of action, and may selectively deplete specific subpopulations of microbes [154–156]. Even antibiotics from the same class can induce different and specific effects on the microbiota [157]. Specific drug, route, spectrum and concurrent disease [113,157], as well as interactions with other non-antibiotic medication and polypharmacy [158], are just some of the factors that make the impact of antibiotic treatment highly individualised and complex.

Suggested treatment for nodular-bronchiectatic MAC-PD, is a 3-drug regimen including a macrolide (e.g., azithromycin, ethambutol and rifampicin), with intravenous amikacin commonly prescribed in addition to the 3-drugs for cavitary, severe or refractory disease [2,15]. Treatment for *M. abscessus*-PD is more complex and variable than that for MAC-PD. The optimal drugs, regimens and duration for treatment of *M. abscessus*-PD are not pre-defined, and rather antibiotic choices are informed by mutational or inducible macrolide resistance patterns [2]. Given the substantial antibiotic burden, we hypothesise that NTM-PD therapy induces significant gut dysbiosis.

Inference of the impact of commonly used antibiotics in disease contexts other than NTM-PD may provide first insights. However, of the drugs used in NTM-PD treatment, to date, there is only information on azithromycin used independently. Azithromycin exposure has been associated with a significant decrease in gut microbial diversity in other disease contexts [155,157,159], and has been negatively correlated with presence of *Actinobacteria* [155,159] and *Bifidobacterium* [157,159]. These changes in microbial diversity and taxonomy are consistent with the broad-spectrum activity of azithromycin. In the context of the GLA and its role in NTM-PD, it is important to consider that antibiotic-associated microbial dysbiosis has been linked to downstream metabolic and immunological changes that may cause or exacerbate disease [155,157,160]. Metabolically, azithromycin exposure in healthy individuals resulted in *Bifidobacterium* depletion which was associated with increases in C-peptide, leading to reduced microbiota-associated glucose metabolism and reduced SCFA biosynthesis [157]. The same study identified azithromycin exposure was correlated with a significant decrease in serum pro-inflammatory cytokines, specifically monocyte chemoattractant protein (MCP)-1 and IL-5 in healthy individuals [157]. Azithromycin has also been reported to attenuate airway inflammation and reduce inflammatory markers in an allergic asthma mouse model [161], as well as to exert anti-inflammatory effects in the lungs of humans with asthma [162]. While these studies suggest possible azithromycin-induced perturbations, the extent, nature, and downstream inflammatory effects of these changes must be independently and thoroughly investigated in NTM-PD, as the impact of antibiotic perturbations may differ across disease states. For example, in bronchiectasis patients treated with erythromycin (which has a similar mechanism of action to azithromycin), significant alterations in microbiota composition were only observed in individuals who did not have an airway infection dominated by *P. aeruginosa* [113]. We hypothesise that similar variability could exist between NTM species, highlighting the need for future studies to examine both drug and species-specific effects on the microbiota. Further research is needed to clarify how azithromycin and other antibiotics used in NTM-PD, both individually and as multi-drug therapy, affect the gut microbiota. The impact of antibiotic treatment on the lung microbiota remains underexplored, both in respiratory diseases and NTM-PD, despite evidence suggesting microbial disruption [113]. Investigating antibiotic effects on both the gut and lung microbiotas, and their potential cross-talk via the GLA, may offer valuable insights into NTM-PD pathophysiology.

To our knowledge, there is one study to date that documents the impact of antibiotic treatment on the gut microbiota in NTM-PD; a study in 27 *M. abscessus*-PD patients with faecal microbiota analysed at baseline, 2 weeks and 6 months of antibiotic treatment (25 of 27 patients on a treatment regimen of azithromycin + amikacin + imipenem/cefoxitin ± clofazimine) [136]. This study provides a clinically relevant perspective by comparing microbiota signatures of *M. abcessus*-PD patients who are treatment-‘responders’ (defined as negative mycobacterial sputum culture at 2 weeks of treatment) to ‘non-responders’ (persistent positive mycobacterial culture at 2 weeks) [136]. The ‘responders’ group showed a significant decrease from pre-treatment baseline faecal microbiota diversity by 2 weeks of antibiotic treatment, and were found to have increased *Enteroccocus* abundance at week 2 [136]. ‘Non-responders’ displayed relative stability in diversity from pre-treatment to 2 weeks and continuing stability at 6 months of antibiotic treatment [136]. Diversity measures at 2 weeks and 6 months were significantly lower in the ‘responders’ group than ‘non-responders’ group [136]. ‘Non-responders’ were also found to have increased abundance of *Eubacterium hallii* group at baseline compared to ‘responders’ [136]. Given the long duration of NTM-PD treatment [2,3,163], determining treatment-responsiveness at 2 weeks is relatively early. However, 14 of the 15 treatment-responders at 2 weeks remained culture negative at 6 months and only four patients displayed a different outcome at 6 months compared to 2 weeks [136]. As the antibiotic regimens in the two patient groups were not significantly different, it was hypothesised that non-responders may have a host-dependent mechanism underlying their treatment unresponsiveness and relatively stable gut microbiota diversity [136]. These observations encourage future studies to assess whether relative stability of microbial diversity following commencement of treatment could be an early indicator for treatment non-responsiveness, or whether taxonomic biomarkers, such as *Enteroccocus* abundance or *Eubacterium hallii* group may predict a patient’s favourable or unsuccessful antibiotic treatment outcome. Such early indicators would hold great potential for early stratification of patients to pursue alternative treatment options. Given the gut microbiota’s potential influence on antibiotic pharmacokinetics, the cyclical relationship between the microbiota and treatment responsiveness may benefit from therapeutic drug monitoring alongside microbiota composition analysis in the future. Differences between *M. abscessus* subspecies (*massiliense* or *abscessus*) [136] and extension to other NTM-PD causative organisms [137,151] should also be considered, to assess potential for universal, early biomarkers of NTM treatment success or failure.

Recovery dynamics following antibiotic treatment—beyond the immediate perturbation—are crucial, as prior antibiotic exposure may influence susceptibility to future infections [154]. Initial and long-term dysbiosis post-treatment cessation depends on the degree of perturbation, the breadth of spectrum of antibiotics, microbial resistance, and the capacity of the microbiota to re-establish equilibrium following perturbation [164]. Reduction in microbial diversity can favour re-colonisation with antibiotic-resistant pathogens [160]. Recovery rates following antibiotic treatment in other diseases are highly variable, from as little as a few weeks, to persistent perturbation up to 14 months post cessation of antibiotic treatment [165,166]. In NTM-PD, studies to date have not addressed long-term gut microbiota composition following treatment. This research will be complicated by the high rates of co-infection with respiratory pathogens in individuals with NTM-PD [167,168]. Antibiotic treatment of these co-infections may cause dysbiosis, a factor that warrants consideration in both research and clinical settings. We hypothesise that the high rates of reinfection and recurrence of disease in NTM-PD may relate, at least in part, to the dysbiosis associated with long-term antibiotic use. Understanding the impact of both NTM-PD-specific antibiotic therapy and treatment for respiratory co-infections on the microbiome is essential, including how the microbiome recovers post-treatment and how these recovery dynamics may influence the risk of relapse or reinfection.

## Conclusions

The microbiome undoubtedly plays a role in health, infection and disease; however substantial knowledge gaps remain regarding its interaction with NTM-PD. It has the potential to influence multiple aspects of the disease, including susceptibility, progression, treatment response, and reinfection or relapse. Host susceptibility factors such as impaired mucociliary clearance and microaspiration, commonly observed in NTM-PD patients, may contribute to microbiota disruption, indicating a plausible link between physiological vulnerability and dysbiosis.

Given the growing recognition of the GLA in other respiratory diseases, investigations into both the gut and lung microbiota may offer valuable insights into the pathophysiology of NTM-PD. To date, studies on the lung microbiota in NTM-PD have been limited with inconsistent findings, likely due to methodological variability. Even fewer studies have examined the gut microbiota in NTM-PD. Further research is needed to characterise potential gut dysbiosis and to investigate how gut microbiota composition interacts with immune responses, the lung microbiota and the overall control of NTM-PD. Future microbiota studies, both in the gut and lung, should endeavour to incorporate larger and better-stratified cohorts, with careful consideration of factors such as causative NTM species, disease severity and radiographic phenotype, and exacerbation states. These variables are essential for improving resolution and interpretability of microbiome data in the context of NTM-PD.

There is a clear need, not just in the context of NTM-PD, to develop a better understanding of the short and longer-term effects of antibiotics on microbial communities at different anatomical sites. Given the individual nature of antibiotic–microbiome interactions, targeted investigation into the effects of drugs and treatment regimens used for NTM-PD and their impact on microbiome recovery is urgently needed.

Ultimately, the gut and lung microbiotas may hold key insights into the mechanisms underpinning the complex nature of NTM-PD. A deeper understanding of microbial dynamics and host responses across these sites could help determine whether microbiome manipulation might prevent disease development and progression, minimise the adverse impact of antibiotic therapy, support post-treatment recovery, and reduce the risk of relapse or reinfection. Such insights have the potential to transform the clinical management of this challenging disease.
